# Computing a ranking network with confidence bounds from a graph-based Beta random field

**DOI:** 10.1098/rspa.2011.0268

**Published:** 2011-08-03

**Authors:** Hsieh Fushing, Michael P. McAssey, Brenda McCowan

**Affiliations:** 1 Departments of Statistics, University of California, Davis, One Shields Avenue, Davis, CA 95616, USA; 2 Department of Mathematics, Vrije Universiteit Amsterdam, De Boelelaan 1081a, 1081 HV Amsterdam, The Netherlands; 3 Population Health & Reproduction, University of California, Davis, One Shields Avenue, Davis, CA 95616, USA

**Keywords:** Beta random field, information transitivity, nonlinear ranking hierarchy, paired comparison, rhesus macaque

## Abstract

We address two largely overlooked, fundamental issues in computing a ranking hierarchy within a society: which information in the network is relevant, and what effect chance has on the hierarchy. To properly account for uncertainty from limited data, we construct a random field in a matrix form having entry-wise posterior Beta distributions based on a graph of pairwise conflict outcomes. To evaluate relevant network information using information transitivity, another random matrix of synthesized transitive dominance odds is computed collectively along observed dominance paths. These two matrices are coupled together to fuse both direct and indirect dominance information. An ensemble of realizations of this fused random matrix facilitates an ensemble of optimal ranking networks by means of simulated annealing. Conditional statistical inferences regarding network features are derived, manifesting the effect of uncertainty. Our computational approach is suitable for large graphs of pairwise conflict outcomes, and can accommodate tremendous data heterogeneity—a typical feature in such studies. We also demonstrate the infeasibility of the classical maximum-likelihood approach, and expose the mechanistic flaws that stem from completely ignoring relevant information residing in the graph. We analyse two real datasets of decisive conflict outcomes, the first involving college football teams, and the second involving an adult rhesus macaque society in captivity.

## 1. Introduction

Consider a society of individuals or organizations among whom many pairwise conflicts (or competitions) are observed over a period of time. The decisive outcomes of these conflicts, in terms of which is identifiable as the winner and which is the loser, carry information about the hierarchy of the society and the relative ranking of individuals and groups within that hierarchy. A collection of pairwise conflict outcomes resulting from such a study is naturally represented by a weighted directed graph. The vertices (or nodes) of this graph represent all subjects that were observed to have participated in at least one pairwise conflict with a decisive outcome. We assign a direction and a weight for the edge between each pair of nodes based on the observed conflict outcomes between the corresponding pair of subjects. The direction represents the evident dominance of one subject over the other, while the weight represents the strength of evidence for that dominance. The most essential advantage of this graphical representation of the pairwise data is the easy visualization of the data structure from a network perspective. That is, not only can we immediately detect the extent of the data heterogeneity, but more importantly we can compute the network structure for the entire system of nodes.

For example, in the USA, the top 25 National Collegiate Athletic Association (NCAA) Division I-A college football teams are assigned ranks throughout the season based on an average of computer-generated rankings and the outcomes of expert opinion polls. The final ranking at the end of the regular season is used to determine which two teams are eligible to compete for the national championship and which teams will participate in various bowl games. The principal criteria for the rankings are the outcomes of the games, in terms of wins and losses. However, the 120 teams in this division generally play only about 13 games in a season, some of which involve opponents outside the division. Hence, a team's winning percentage is not sufficiently informative about its strength. It is necessary to base a team's rank in part on the performance of that team's opponents in their games, so that beating successful teams carries greater weight. Thus, this endeavour requires a complex inter-related structure, making the computation of the overall ranking especially challenging.

As a second example, the California National Primate Research Center (CNPRC) at the University of California, Davis, houses multiple outdoor captive groups of rhesus macaques. Researchers observe the interactions among the macaques in each cage and record data regarding conflicts between them. Such conflicts include all aggressive interactions that had a decisive outcome. For example, an interaction where an initiator threatens a recipient and the recipient runs away is counted as a win for the initiator and a loss for the recipient. Other aggressive behaviours include lunging, chasing and biting. These observed behaviours demonstrate an intrinsic social hierarchy wherein some individual macaques and family groups (matrilines) possess a higher dominance status. In Fushing *et al.* (2011), a new approach is developed to infer this hierarchy based on the conflict data. Although this approach proved quite useful to primatologists and confirmed their hypotheses about the macaque social structure, the method had several shortcomings which hindered its ability to effectively accommodate other settings, such as the NCAA rankings.

The general goal of a paired-comparison study is to transform an empirical directed graph based on these kinds of data into a ranking hierarchy or network. Two fundamental issues involved in such a task are:

*Question* Q1: In the directed graph, where is the relevant information contained?

*Question* Q2: How can we properly assess the uncertainty of network features?

From the information aspect of Q1, a record of pairwise conflict outcomes provides only direct and local information for the global ranking hierarchy. The locality of this direct information alone is not sufficient to construct a ranking network without assuming a putative global structure. But if the edges of a graph are linked in a certain fashion, they serve as evidence that contains indirect local information about ranking as well. This concept heuristically says that if dominance information is perceived to transmit through an edge as if through a unidirectional noisy channel—as in signal processing or communication—then we should be able to find an abundance of valuable dominance information between any two nodes indirectly. Specifically, we intuit that dominance information transmits from one edge through other edges which have the same dominance direction, although in doing so the information content is attenuated owing to the addition of noise. This concept is called *information transitivity*. For example, if we know that *A* dominates *B* and *B* dominates *C*, then we can infer that *A* dominates *C*. We further intuit that the information in one edge cannot be transmitted through any other edge whose dominance direction is contrary to its own direction. That is, if we know that *A* dominates *B* but *B* is dominated by *C*, then we can infer nothing about whether or not *A* dominates *C*.

To the best of our knowledge, the information transitivity induced by such a graph has not yet been explored in the paired-comparison literature. We make use of this concept to extract indirect dominance information through a sequence of edges with the same direction, which we call a *dominance path*. Let the observed empirical graph be formally denoted by 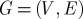, where *V* and *E* are the collections of subject nodes and of directed edges, respectively. The nodes are arbitrarily labelled 1,…,*N*, while each directed edge is equipped with weights (*c*_*ij*_,*c*_*ji*_), where *c*_*ij*_ is the number of observed conflicts between subjects *i* and *j* in which subject *i* was the winner, and *c*_*ji*_ is the number in which subject *j* was the winner. An edge is present between nodes *i* and *j* if *c*_*ij*_+*c*_*ji*_>0. The sign of its direction from *i* to *j* is positive (+) if *c*_*ij*_>*c*_*ji*_ and negative (−) if *c*_*ij*_≤*c*_*ji*_. A connected path in *G* from onset node *i* to offset node *j* is considered a dominance path if node *i* is linked to node *j* by successive +-directional edges. All nodes on a dominance path, excluding the onset and offset nodes, are called intermediate nodes.

Intuitively, each dominance path implies a certain degree of empirical transitive dominance of the onset node over the offset node. If there is a large collection of non-overlapping dominance paths linking the same pair of onset and offset nodes, then we expect a large degree of empirical transitive dominance of the onset node over the offset node (here two paths are non-overlapping if they do not share any common edge, i.e. any common intermediate node). Of course, in any real dataset there are inevitably many conflicting dominance paths, leading to varying degrees of uncertainty about the relative ranks of the subjects being studied. We expect that the use of information transitivity along dominance paths will have a great impact on the success of the ranking task while providing a resolution to both Q1 and Q2. The reason for this impact is as follows.

The endeavour to construct a ranking network always faces the challenging issue: how can we transform local information into global information? The major challenges come from the fact that, if a classical version of transitivity is imposed to provide the means of going from a local to a global ranking network, an empirical directed graph usually cannot be turned into a ranking network in a straightforward manner owing to the prevalence of divergent evidence. This divergent evidence is commonly manifested through many forms of motifs or patterns in the data graph. For instance, one prospective high-ranking subject may lose one or more conflicts to a prospective low-ranking subject. There generally exist many prospective ranking patterns which are circular. The underlying mechanism of classical transitivity, such as the weak or strong stochastic transitivity found in Davidson & Solomon (1973), are like mathematical logic rules. They are too rigid to handle the aforementioned divergent information, while our use of information transitivity can easily adapt to such information.

Nevertheless, the classical transitivity constraint is commonly imposed either implicitly or explicitly in constructing ranking hierarchies. Under this constraint, the ranking task is performed routinely in many aspects of human society, from NCAA college football team rankings to business decision-making, from individual choices to corporate plans, and from governmental policy to academic research. So would it not be legitimate to question the authenticity of these applications when there is divergent information embedded in almost all real-world datasets involving conflict outcomes? Considering such a wide spectrum of applications, and keeping the authenticity of the question in mind, it is surprising that in the literature the chief way of avoiding the divergent information patterns is to make use of a linear dominance model, such as the popular one proposed by Bradley & Terry (1952), which can be traced back to Thurstone (1927). This linearity modelling assumption uses only the direct information from decisive conflict outcomes to construct a likelihood function, and wishfully thinks that the likelihood contains all the information needed for constructing the ranking network.

The popularity of this approach is owing to a great extent to the illusion that it is free from the necessity of accommodating the classical transitivity into the likelihood function, which would indeed be very difficult if not impossible. Specifically, let *C* denote the matrix whose off-diagonal elements consist of the data *c*_*ij*_. The linearity modelling assumption postulates that subject *i* is equipped with some intrinsic dominance potential *d*_*i*_ imposed by nature, which dictates which individuals in the society will submit to him and to which individuals he will submit. That is, if *d*_*i*_>*d*_*j*_ then subject *j* will submit to subject *i* in any confrontation. Under this approach, one arbitrarily sets *d*_*N*_=0 to establish a baseline dominance potential, and lets **d**=(*d*_1_,…,*d*_*N*−1_). Since subject *N* is not necessarily at the bottom of the hierarchy, it is possible for *d*_*i*_<0 for one or more subjects. All that ultimately matters is the order of the dominance potentials, so the choice of baseline is not important. Then the likelihood function is calculated as

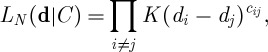
where the logistic version of the Bradley–Terry model sets

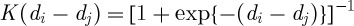
as the kernel that transforms the difference between dominance potentials into a probability of winning. Therefore, the full likelihood involves *N*−1 parameters. The resulting ranking is obtained by simply reordering the maximum-likelihood estimate (MLE) 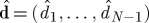 from largest to smallest.

We immediately recognize the potential curse of dimensionality that will cause the MLE to be very unreliable. But beyond that, in this setting, there is neither a need to deal with the inevitable divergent information, nor to accommodate classical transitivity. All difficulties are seemingly resolved by this modelling setup and the likelihood approach. Many practitioners and researchers are so allured by the convenience and computational simplicity as to believe that the likelihood function contains all the information embedded in the data set, and that MLE takes care of all the divergent patterns that appear in the data graph. We claim this assurance is not valid under this network setting.

This is indeed a misconception in network-related analysis. There is no way that any modelling structure, such as the linearity in ranking, can model away the difficulties in accommodating the empirically observed divergent information and simultaneously achieve the nearly impossible task of retaining the classical transitivity constraint. The derived likelihood function simply ignores the divergent information and provides a compromised answer in a heavy-handed but an unknown fashion. Our concern becomes even more profound when the data involve considerable heterogeneity, such as when a sizable proportion of pairs have *c*_*ij*_=*c*_*ji*_=0 and prospective high-ranking subjects have almost no direct contact with very low-ranking ones. We illustrate in the next section that the MLE approach also produces counterintuitive ranking results. Through the two examples, we motivate our use of information transitivity and the development of a methodology that can resolve the central issues of deriving ranking networks in most paired comparison studies and applications.

The general idea of our approach is this: we develop a random field consisting of random variables *P*_*ij*_ with posterior Beta distribution Be(*c*_*ij*_+1,*c*_*ji*_+1) on each edge linking node pair (*i*,*j*). That is, *P*_*ij*_ is a possible value of the probability that subject *i* is dominant over subject *j*, based on the outcomes of the observed conflicts between them. We regard this as the *dominance probability* of *i* over *j*, with corresponding direct *dominance odds* 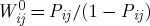. This random field allows us to account for the uncertainty inherent in the data. We further derive a scheme to compute the dominance odds along each dominance path by mimicking a signal passing through a noisy channel. A dominance path with no intermediate nodes provides the most direct and least noisy information about the dominance odds, while paths with one or more intermediate nodes provide indirect and noisier information through the instrument of transitivity. Hence, a collection of non-overlapping paths produces a collection of mutually independent direct and indirect sources of information about the dominance odds of *i* over *j*. The collection of the corresponding indirect dominance odds computed via transitivity is multiplied together to produce the synthesized indirect dominance information. It is then coupled with the corresponding direct dominance odds to produce an enhanced version 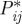 of the random variable *P*_*ij*_, which represents the dominance probability of onset node *i* over offset node *j*. The random variables for all node pairs are collected together to form a random matrix P* of enhanced dominance probability.

Furthermore, to go from local to global information while simultaneously evaluating the effect of uncertainty, we generate a large ensemble of realizations of this random matrix. Upon each realization, we use a global optimization technique—the Simulated Annealing Algorithm—to find the optimal ranking coordinates as a realization of the ranking network. Finally, this ensemble of optimal permutations of *i*=1,…,*N* forms the basis for statistical inference on the ranking network, including a mean-field version of the ranking network for point estimation of ranking coordinates. We may then provide a confidence set for any network feature of interest with a desired level of confidence.

Consequently, our development conceptually recognizes that the local information regarding the dominance of one subject over another makes its contribution to the global ranking network through its connected neighbouring subjects. We particularly focus on the whole collection of dominance paths up to a chosen length between a focal pair of onset and offset nodes. Therefore, our algorithmic statistical inference is conditional, given the collection of chosen dominance paths. In this way, our ranking network jointly reflects the locality of the targeted global structure.

We demonstrate our approach through the analysis of two real datasets of conflict outcomes. The first involves the 120 NCAA Division I-A college football teams in the USA during the 2010 regular season. The second concerns a colony of captive adult rhesus macaques at the CNPRC in 2008–2009. Both computed ranking hierarchies correspond well with the expectations of experts.

## 2. Motivation and illustrating examples

We impose two assumptions as the backbone of our developments in this paper:

A1 We have independence among the collection of conflict outcome data.
A2 Non-overlapping dominance paths independently provide degrees of stochastic transitive dominance potential.

Assumption A1 is made purely for computational feasibility. We are aware of possible local dependencies, or even global dependence (such as behaviour cascades), embedded in the directed data graph (Mézard & Montanari 2008). However, dealing with dependence-induced complexity is beyond the scope of this endeavour. Assumption A2 is a device to tailor scattered pieces of local information together. Neither ‘weak’ nor ‘strong’ stochastic transitivity is used here (Davidson & Solomon 1973). Nonetheless, in the next section, we develop the concept of information transitivity, and its result, called *filtered transitive dominance*, to extract indirect information that can enhance or dilute the direct dominance information provided by the pairwise conflict outcomes. In this section, we illustrate why the MLE produces counterintuitive results through simple examples, and at the same time we motivate resolutions for Q1 and Q2 under assumptions A1 and A2.

For illustration, consider the following two examples: (1) three nodes with highly heterogeneous conflict outcome records; (2) four nodes with uniform single conflict outcome records. Both cases result in a counterintuitive ranking order under the Bradley–Terry model with a logistic probability function.

**Example 2.1**

**Figure 1.**
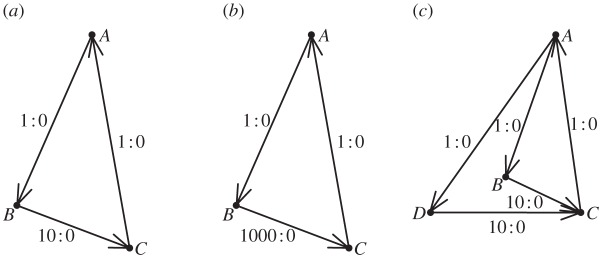
Three examples of conflict networks. (*a*,*b*) Networks of three nodes and (*c*) four nodes with edge weights and directions based on decisive conflict outcomes.

Consider three nodes labelled *A*, *B* and *C*, with corresponding dominance potential parameters *d*_*A*_, *d*_*B*_ and *d*_*C*_=0. Recall that *c*_*ij*_ is the number of observed conflicts in which node *i* defeated node *j*. Consider the hypothetical observation record: *c*_*AB*_=1, *c*_*BA*_=0, *c*_*AC*_=0, *c*_*CA*_=1, *c*_*BC*_=10 and *c*_*CB*_=0, as graphically portrayed in figure 1*a*. Under the Bradley–Terry model, the likelihood is calculated as

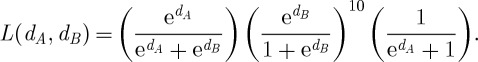
The MLE is computed to be 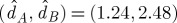, with the maximum log-likelihood value equal to −3.79. The point estimate of the odds of node *A* dominating node *C* is thus equal to 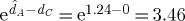. This result is counterintuitive. The reason is as follows: given that it is almost certain that node *B* dominates node *C*, the one win of node *A* over node *B* logically can be regarded as ‘a fraction of one win’ of node *A* over node *C* via the idea of transitivity. That is, at the very least, nodes *A* and *C* should have virtually equal odds for winning one and losing one to each other. This heuristic idea is also supported by the following computations. With data *c*_*AB*_=1 and *c*_*BA*_=0, the posterior mean of the dominance probability of node *A* over node *B*, based on the posterior distribution Be(2,1) derived from the prior distribution Be(1,1), is calculated to be 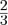. Likewise, the posterior mean of the dominance probability of node *B* over node *C*, based on the posterior distribution Be(11,1), is 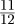. We can conservatively compute that the indirect filtered transitive dominance odds of node *A* over node *C* is equal to

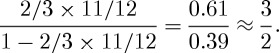
The direct dominance odds of node *A* over node *C*, based on the single loss of node *A* to *C*, is calculated to be 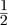. Then combining the direct and indirect dominance odds into the resulting dominance odds of node *A* over node *C* is only 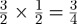. Hence, our intuitive computations result in an estimate of the dominance odds that is strikingly different from the one derived from maximum-likelihood estimation.

One can ordinarily use the observed Fisher information matrix to obtain a CI for the MLE, provided there are sufficiently many observations to justify a normal approximation. A naive normality assumption in this example would yield a 95% CI for *d*_*A*_ which includes zero, so that the conflict with our intuitive result is seemingly resolved. However, there are far too few observations to support using a normal approximation for such an inference.

Switching to the network depicted in figure 1*b*, where *c*_*BC*_=1000, the computed MLE is 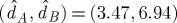. Consequently, the dominance odds for node *A* over node *C* is *e*^3.47^=32.14. However, the 95% CI for *d*_*A*_ based on the observed Fisher information matrix again includes zero, and now there should be enough observations to justify a normal approximation. Yet the 95% CI for *d*_*B*_ includes zero as well, even though *B* is clearly dominant over *C*. Hence, the likelihood approach is unable to give precise parameter estimates in this example. Meanwhile, using the same heuristic demonstrated above, the dominance odds of node *A* over node *C* is

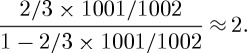
Therefore, by combining this indirect dominance odds with the direct dominance odds, the resulting estimate of the dominance odds of node *A* over node *C* is 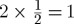; that is, nodes *A* and *C* are equal in rank. Heuristically speaking, the one win of node *A* over node *B* is regarded fully as a win over node *C*, with no discounting effect as in the case pertaining to the data in figure 1*a*. This is a much more satisfying result than that obtained under the likelihood approach.

On the other hand, if there exists an extra dominance path that provides another independent source of filtered transitive dominance odds, such as is portrayed in figure 1*c*, we can heuristically compute the indirect dominance odds of node *A* over node *C* as

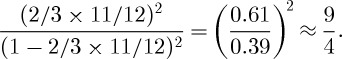
Then combining this indirect dominance odds with the direct odds, the resulting dominance odds of node *A* over node *C* is 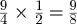. Hence the dominance probability of node *A* over node *C* is 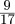 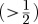. These series of Bayesian computations give us estimates of the dominance odds which match our intuition very well. At the same time, we demonstrate the importance of incorporating information through filtered transitive dominance. In contrast, we also illustrate that the likelihood approach under a linearity assumption within the Bradley–Terry model does not work as well as we expected. This computational approach via the posterior mean, first developed in Fushing *et al.* (2011), is rather conservative in extracting indirect dominance information relative to the improved approach based on the Beta random field which is developed in the next section.

**Example 2.2**

**Figure 2.**
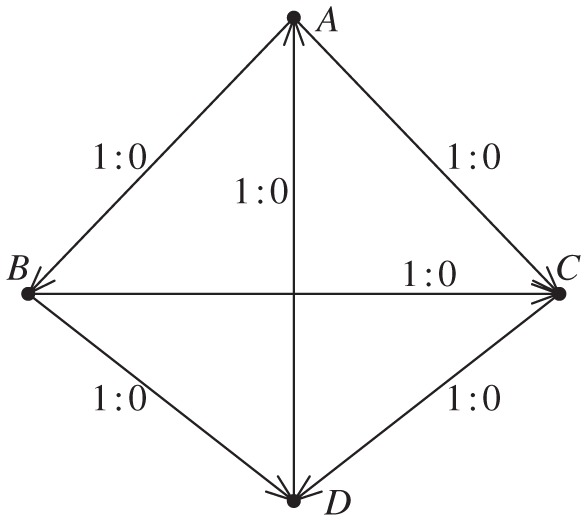
Example of conflict network. Network of four nodes with edge weights and directions based on decisive conflict outcomes.

In the next example, the ranking uncertainty is more evidently illustrated than that in the previous example. Consider a set of conflict outcome data, as portrayed graphically in figure 2, as follows: *c*_*AB*_=1, *c*_*BA*_=0, *c*_*AC*_ = 1, *c*_*CA*_=0, *c*_*BC*_=1, *c*_*CB*_=0, *c*_*BD*_=1, *c*_*DB*_=0, *c*_*CD*_=1, *c*_*DC*_=0, *c*_*AD*_=,0 and *c*_*DA*_ = 1. The likelihood function of (*d*_*A*_,*d*_*B*_,*d*_*C*_) under the Bradley–Terry model (with *d*_*D*_=0), with a logistic probability function, is calculated as

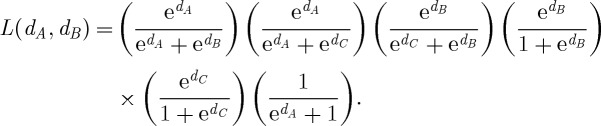
The computed MLE is 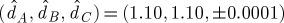, with the maximum log-likelihood value equal to −3.64. The ± is owing to the fact that the likelihood function around the vicinity of (1.10,1.10,0.0) is rather flat and gives rise to an estimate that is imprecise around zero. However, the MLE seems to indicate strongly that nodes *A* and *B* dominate nodes *C* and *D*, despite the sparse and conflicting evidence. This result can hardly be regarded as reliable. Since there are only six observations, it is pointless to derive CIs for the parameters using the observed Fisher information matrix.

In contrast, the approach using a Beta posterior mean does not provide an assertive dominance odds estimate for *any* pair of nodes. This inconclusive result points to an important issue in any paired comparison analysis: the ranking uncertainty is essential and needs to be accommodated. This paper proposes a computational approach to bring out such kinds of uncertainty not only for any pair of nodes, but also for any empirical global features or patterns embedded within the whole ranking network. The heuristic idea and scheme are illustrated through example 2. For each pair of nodes, we assume that the prior winning probability, say *P*_*ij*_, of one node over the other follows the standard uniform distribution, i.e. the Be(1,1) distribution. With pairwise conflict outcomes (*c*_*ij*_,*c*_*ji*_) between nodes *i* and *j*, and *n*_*ij*_=*c*_*ij*_+*c*_*ji*_, the corresponding posterior distribution of *P*_*ij*_ is Be(*c*_*ij*_+1,*c*_*ji*_+1), with posterior mean (*c*_*ij*_+1)/(*n*_*ij*_+2). This pairwise Bayesian structure with a posterior distribution avoids infinite dominance odds when there are no losses and a positive number of wins, while keeping the odds equal to one when there are no losses and no wins. The direct dominance odds of node *i* over node *j* is then denoted by 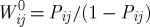.

With the above pairwise posterior distribution structure, we can derive a filtering scheme for transitive dominance as follows. Let 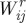 denote the indirect dominance odds of node *i* over node *j* via information transitivity of *order* *r*, based on *all* independent dominance paths having *r* intermediate nodes. Meanwhile, let 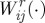 denote the indirect dominance odds of node *i* over node *j* based on the specific dominance path through the *r* intermediate nodes specified within the parentheses. To maintain independence of dominance paths, edges on any dominance path between two nodes cannot appear in any other dominance path between the same two nodes. We argue in a supplemental file that the inclusion of such paths in our computation of indirect dominance odds is not necessarily a liability, but for the moment we confine our analysis to independent paths.

For example, consider the winning path from node *A* to *B* and node *B* to *D* in figure 2, and denote this dominance path by *σ*(*A*,*B*,*D*). Thus, node *B* is the only intermediate node between onset node *A* and offset node *D* on this dominance path. The random variables representing the dominance probability of node *A* over node *B* and the dominance probability of node *B* over node *D*, *P*_*AB*_ and *P*_*BD*_, respectively, are filtered—or diluted—and transformed into the transitive dominance odds of node *A* over node *D* through single intermediate node *B* by the following scheme:

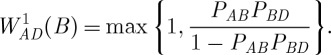
When there are two or more intermediate nodes, the form of this expression is extended. For example, along the dominance path *σ*(*A*,*C*,*B*,*D*), the transitive dominance odds of node *A* over node *D* through intermediate nodes *C* and *B* is

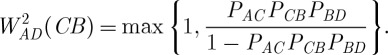
This filtering is conservative. In order to obtain a value of 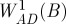 larger than 1, both *P*_*AB*_ and *P*_*BD*_ must be much larger than 0.5, so that their product exceeds 0.5. Likewise, to obtain a value of 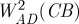 larger than 1, the product *P*_*AC*_*P*_*CB*_*P*_*BD*_ must exceed 0.5. From this perspective, our filtered transitive dominance does not follow weak stochastic transitivity (Davidson & Solomon 1973). Also, choosing the maximum is a device designed to retain only the directional dominance without imposing a penalty. Consequently, this indirect dominance odds of node *A* over node *D* via the filtered transitive dominance through path *σ*(*A*,*B*,*D*) is a random variable taking a discrete value equal to 1 with high discrete probability and a continuous value following a density. Hence, the uncertainty can be well-expressed through the distribution of this random variable 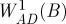. Furthermore, in order to evaluate the complete set of filtered transitive dominances, we must look for all independent (non-overlapping) dominance paths of node *A* over node *D* via different intermediate nodes among all subgraphs sharing the edge between nodes *A* and *D*.

To complete evaluating the dominance odds of node *A* over node *D*, we must seek all directed dominance paths between the two nodes. So for the indirect dominance of node *A* over node *D*, we observe three paths on the directed graph displayed in figure 2: *σ*(*A*,*B*,*D*), *σ*(*A*,*C*,*D*) and *σ*(*A*,*C*,*B*,*D*). This latter path through intermediate nodes *C* and *B* overlaps with both *σ*(*A*,*B*,*D*) and *σ*(*A*,*C*,*D*), so we might disregard it outrightly. However, even if we do not, this dominance path is likely to be filtered, since *P*_*AC*_*P*_*CB*_*P*_*BD*_ is very unlikely to exceed 0.5 in this example. Hence, the resulting dominance-odds random variable *W*_*AD*_ is the product of four random dominance odds: 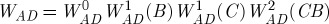. The distribution of *W*_*AD*_ will be analytically derived and numerically evaluated in the next section.

Similarly, we have the following dominance odds derivations:

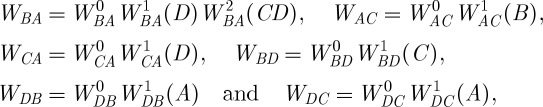
while 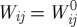 for all remaining pairs (*i*,*j*).

Intuitively, based on the six distributions of *W*_*ij*_, it is rather difficult to conceive that there could possibly be evidence for linearity in ranking among the four nodes, as strongly indicated by a dominance potential scheme such as 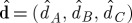 under the Bradley–Terry model. Many ranking features are obscured by significant levels of uncertainty. We believe that this computational approach, using a Beta random field, is far more realistic.

## 3. Beta random field and filtered transitive dominance odds

We construct a Beta random field upon a network in the following fashion. We have denoted the dominance probability of node *i* over node *j* by the random variable *P*_*ij*_, and have shown that the posterior distribution of *P*_*ij*_, given *c*_*ij*_ and *c*_*ji*_, is Be(*c*_*ij*_+1,*c*_*ji*_+1). With such a Beta posterior distribution coupled with a + or − direction on each edge of a network, we say that the network is equipped with a *Beta random field*.

**Figure 3.**
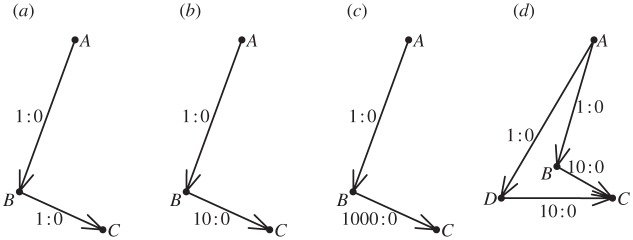
Examples of dominance paths of order 1. Dominance paths from node *A* to *C* through intermediate nodes *B* and *D*, with win–loss ratios.

In this paper, we focus on independent dominance paths of orders 1,2,…, up to some reasonable threshold *R* dependent on the data. Let us first confine our development to the case *R*=1. Let 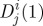 denote the collection of order-1 dominance paths having node *i* as the onset and node *j* as the offset. An order-1 dominance path in 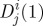 through node *h* is then denoted by *σ*(*i*,*h*,*j*). We illustrate such paths in figure 3. Figure 3*a*–*c* presents a dominance path from onset node *A* to offset node *C* through intermediate node *B*. Each arrow from node *k* to node ℓ is labelled with the ratio of wins *c*_*k*ℓ_ to losses *c*_ℓ*k*_ among observed conflict outcomes between the corresponding individuals. Note that *c*_*BC*_ increases from 1 to 10 to 1000 from figure 3*a* to 3*b* to 3*c*. In figure 3*d* a matching dominance path from node *A* to node *C* through intermediate node *D* has been added to that which is displayed in figure 3*b*.

In general, the filtered transitive dominance odds of node *i* over node *j* through the order-1 path *σ*(*i*,*h*,*j*) is defined as

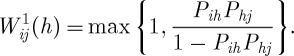
The distribution of the random variable 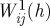 is derived as follows. First, we derive the density of random variable *O*_*ij*_=*P*_*ih*_*P*_*hj*_/(1−*P*_*ih*_*P*_*hj*_). With some labour we arrive at

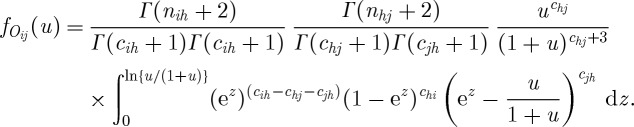
Secondly, we note that the random variable 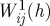 has a discrete part taking the value 1 with probability 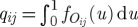 and a continuous density part specified by *f*_*O*_*ij*__(*u*) in the range 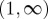.

The random variable of filtered transitive dominance contributed by the collection of order-1 dominance paths from node *i* to node *j* is then calculated as

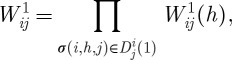
and its distribution can be derived theoretically by employing a logarithmic transform and the mutual independence of the random variables 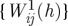. This derivation is not straightforward, and the finite normal approximation is unsuitable since each random variable is a mixture of a discrete part and a continuous part. Although its distribution can be numerically evaluated using *f*_*O*_*ij*__(*u*), applying sampling techniques to simulate and approximate the distribution of 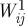 would be far too clumsy, since the form of *f*_*O*_*ij*__(*u*) is quite involved. As an alternative, we evaluate the distribution of 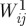 by sampling from a random field. Furthermore, it is intuitive that the distributional form of 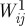 will be critically determined by the size of 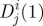. When its cardinality 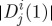 is small, the discrete part of 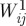 is still significant, since 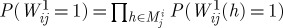 will be small but not insignificant. But when 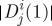 is large, this product will approach zero, leaving only the continuous part, so that 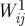 behaves much like a continuous random variable.

**Figure 4.**
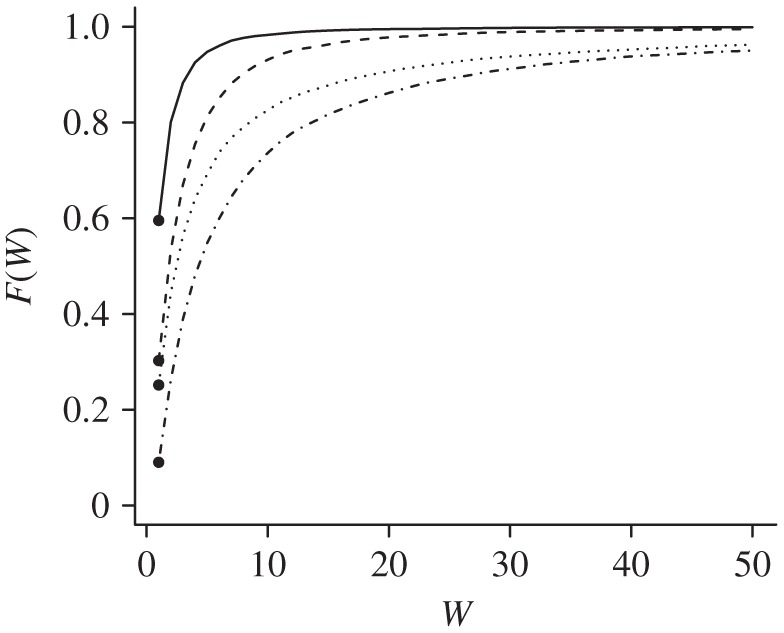
Empirical cumulative distribution functions (CDFs) for *W*^1^_*AC*_. Empirical CDF for 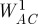 corresponding to the four configurations shown in figure 3*a*–*d*. (*P*(*W*^1^=1) Solid line, 0.5954; dashed line, 0.3026; dotted line, 0.2517; dashed-dotted line, 0.0902).

In figure 4, plots of the empirical cumulative distributions of 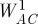 for the four cases displayed in figure 3 are demonstrated. In each case, we draw 10 000 conditional realizations of *P*_*AB*_, given *c*_*AB*_ and *c*_*BA*_, from the Be(*c*_*AB*_+1,*c*_*BA*_+1) distribution. We do likewise for *P*_*BC*_ and in the fourth case, for *P*_*AD*_ and *P*_*DC*_, so that we have 10 000 sets of realizations for each of the four cases. For each set of realizations we compute 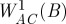 and, in the fourth case, 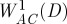. For the first three cases, we have 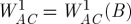, while for the fourth case we have 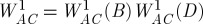. The proportion of the 10 000 computations of 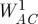 for which its value equals one is the empirical probability 
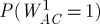
, and is thus an estimate of the discrete part of 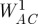. The remaining computations for which 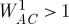 give the empirical probability 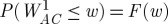 for *w*>1, and is thus an estimate of the continuous part of 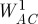. The top-most plot of *F*(*w*) corresponds to figure 3*a*. Below that is the plot of *F*(*w*) corresponding to figure 3*b*, with that corresponding to figure 3*c* below it, and the plot corresponding to figure 3*d* at the bottom. We note that, as we move from left to right in figure 3, the discrete part of 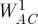 diminishes, so that the filtered transitive dominance between *A* and *C* will contribute more information toward the estimate of the dominance probability *P*_*AC*_.

Let us denote the *N*×*N* random matrix of filtered transitive order-1 dominance odds by 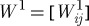. This matrix is based on the Beta random field and is capable of offering the indirect order-1 dominance odds for the whole ranking network. Thus, *W*^1^ is a kind of conditioning statistic given the configuration of the graph. In contrast, the *N*×*N* random matrix 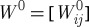, with 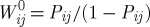, offers the direct dominance odds based on the conflict matrix *C*. Combining the direct and indirect dominance odds, we arrive at the synthesized dominance odds random matrix based on the network graph as

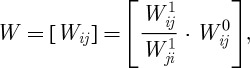
where ⋅ denotes the entry-wise product. While, it is always the case that 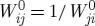, it is not generally the case that 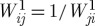. Yet, it is essential that *W*_*ij*_=1/*W*_*ji*_ so that the enhanced dominance probability derived below satisfies 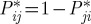. Dividing 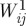 by 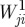 in the computation of *W*_*ij*_ ensures this reciprocal relationship. Moreover, this step incorporates a balance of all conflicting evidence regarding the dominance between node pairs, as when there are dominance paths of various lengths flowing in both directions. Thus, if both 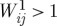 and 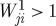, this step incorporates only the relative size of one to the other in the computation of the synthesized dominance odds *W*_*ij*_ and *W*_*ji*_.

As stated above, we may also consider dominance paths of higher order, up to some threshold. Let 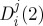 denote the set of all order-2 dominance paths from *i* to *j* through two intermediate teams, such as when node *i* beats node *h*, node *h* defeats node *k* and node *k* beats node *j*. To maintain independence of dominance paths, we must ensure that 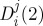 contains no paths which overlap each other nor overlap any paths in 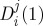. This can create considerable computational complexity, and we argue in the electronic supplementary material that treating all distinct dominance paths as independent, whether or not they overlap, is very unlikely to inflate the indirect dominance odds. We then denote the random variable representing the order-2 dominance odds of node *i* over node *j* through all order-2 dominance paths from *i* to *j* as

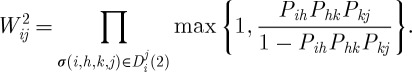
The computational impact of order-2 information transitivity on the indirect dominance is thus even more conservative, as the product *P*_*ih*_*P*_*hk*_*P*_*kj*_ must exceed 0.5. Likewise, we compute the random variable representing the order 3 dominance odds of node *i* over node *j* through all order-3 dominance paths from *i* to *j* as

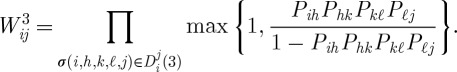


We continue to impute dominance odds via transitivity in this manner through order *R*. The information is then synthesized to produce the random variable *W*_*ij*_ representing the overall odds that node *i* dominates node *j* based on both direct conflicts and indirect dominance through information transitivity up to order *R*, computed as

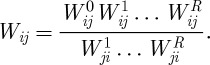
The denominator is designed to ensure that *W*_*ij*_=1/*W*_*ji*_. The matrix *W*=[*W*_*ij*_] then contains the synthesized dominance odds up to order *R* for all node pairs. Now it may be that node *j* has beaten node *i*, even though there is evidence that node *i* dominates node *j* indirectly through various dominance paths of different orders, leading to conflicting information. There may even be dominance paths of various orders from *j* to *i* that produce further conflict. But our method accommodates and integrates such conflicting information to produce a balanced evaluation of the dominance relationships among all pairs of nodes.

It is necessary, of course, to choose a suitable value for the threshold *R*. Finding dominance paths and computing the order *r* odds up to a large value of *R* among all *N*(*N*−1) ordered pairs of nodes would be computationally intensive even for a moderate *N*. But if there are few order-0 and order-1 dominance paths between a majority of node pairs, *R* should be sufficiently large so as to extract enough useful transitive dominance information from the record of conflicts such that a meaningful ranking is possible. We regard the distance from node *i* to node *j* to be the shortest dominance path from *i* to *j*. The diameter of the network is the largest distance among its *N*(*N*−1) ordered node pairs. We may also consider the average distance among the node pairs. To ensure useful ranking information without overtaxing the computations, we suggest that *R* should be chosen at least as large as this average distance, and no larger than the diameter.

Finally, the enhanced dominance probability matrix P* based on *W* is derived as

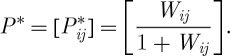
We can then construct the ranking hierarchy based on an optimal permutation of the rows and columns of P*, and make inference based on the distribution of the ranking network. These tasks are performed by applying the algorithm given in the next section. We believe that the effect of chance in the ranking hierarchy is addressed in this fashion for the first time in the paired-comparison literature.

## 4. Computing algorithm

Our computing algorithm is conditional on the observed empirical graph, in the sense that we not only fix the observed conflict outcomes matrix *C*, but also, for each (*i*,*j*) pair, we fix its empirically observed collection of dominance paths. Our algorithm based on the Beta random field takes the following steps:

Step S1: We choose a large integer *K* and a suitable threshold *R*, and create an ensemble of *K* dominance probability matrices conditioned on the conflict matrix *C*. For the *k*th replication, 1≤*k*≤*K*, we generate a random value 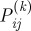 from Be(*c*_*ij*_+1,*c*_*ji*_+1) for all (*i*,*j*) with *i*<*j*, and set 
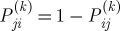
. However, to reduce the variability in the random field, when *c*_*ij*_=*c*_*ji*_=0 we set *P*_*ij*_=*P*_*ji*_=0.5, which is the mean of the corresponding Be(1,1) distribution. Then we follow the procedural steps described in the previous section to derive the matrices *W*^0^,*W*^1^,…,*W*^*R*^, and finally the synthesized dominance odds matrix *W* and the corresponding enhanced dominance probability matrix P^*(*k*)^. Once we have the ensemble 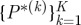, we compute their component-wise average

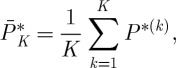
which we call the *mean field*.

Step S2: For each enhanced dominance probability matrix replication in the ensemble 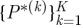, as well as for the mean field 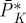, an optimal set of ranking coordinates is individually computed and denoted by *σ*^(*k*)^, *k*=1,…,*K*, and 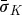, respectively. Ideally, an optimal set of ranking coordinates *σ*^(*k*)^ is a permutation of the node indices (1,2,…,*N*) such that, when the rows and columns of P^*(*k*)^ are permuted according to *σ*^(*k*)^, the resulting permuted matrix 〈P^*(*k*)^〉_*σ*^(*k*)^_ has as little cumulative error as possible. An error occurs whenever one subject is assigned a rank *j* while another subject is assigned an inferior rank *i*>*j*, but the (*i*,*j*) component of 〈P^*(*k*)^〉_*σ*^(*k*)^_ exceeds 0.5. The error is magnified based on the extent to which this component exceeds 0.5, and based on the size of the difference *i*−*j* in their assigned ranks. Hence, an optimal permutation should produce as few entries exceeding 0.5 as possible in the lower triangle of the permuted matrix, and those entries which do exceed 0.5 should be as small as possible and as close to the diagonal as possible. Mathematically, we define the optimal permutation as

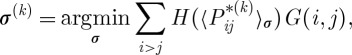
where the minimum is taken over all possible permutations *σ* of (1,2,…,*N*), the loss function *H*(*p*) is a function on [0,1] that takes the value zero for *p*∈[0,0.5] and takes strictly increasing positive values on (0.5,1], and the penalty function *G*(*i*,*j*) increases as (*N*+1−*j*)(*i*−*j*) increases, i.e. as (*i*,*j*) is further from the diagonal. In our computation we use 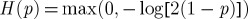, as in Fushing *et al.* (2011), and 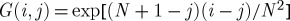. The optimization is carried out by applying the simulated annealing algorithm (Kirkpatrick *et al.* 1983), which requires choosing a suitable neighbourhood system and a scheme for decreasing the temperature. See Fushing *et al.* (2011) for a full description. In general, the cumulative error is substantially lower when the mean field 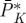 is optimized than is the cumulative error when any individual matrix P^*(*k*)^ is optimized.

Note that the optimization on *K*+1 matrices via simulated annealing can be split up among multiple servers to reduce the computational time. Moreover, if *K* is large, one may choose to optimize only a subset of the matrices among 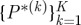. For example, if *K*=1000 one might optimize every tenth matrix and also the mean field. This would yield a mean-field ranking based on all 1000 generations of P*, and 100 sets of ranking coordinates suitable for empirical confidence bounds, as described in the next step.

Step S3: We take the optimal set of ranking coordinates 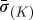, based on optimization of the mean field 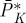, as the point estimate for the ranking coordinates of the network. The confidence set for any network features of interest are then based on the ensemble 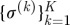. For instance, a 90 per cent empirical CI for the rank of any particular node can be derived by computing the 5th and 95th quantiles of the distribution of that node's assigned ranking among 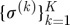.

**Figure 5.**
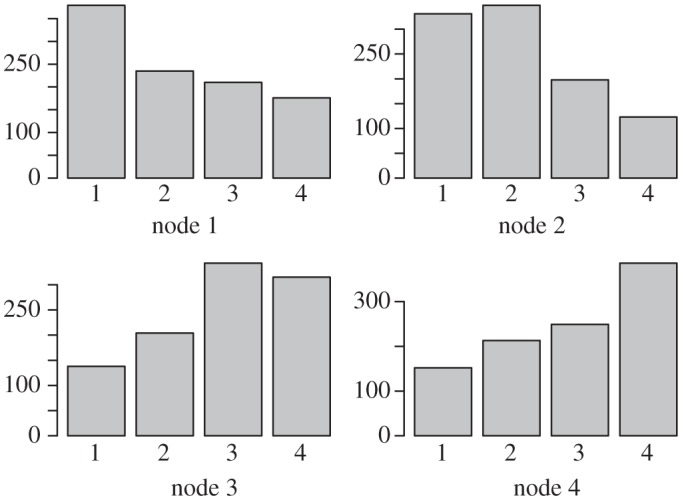
Frequency distribution of rankings. Bar plots for each node of figure 2 indicating the frequency of assignments of that node among each of the four possible ranks among 1000 iterations of our computing algorithm applied to the corresponding network.

We apply this computing algorithm with *K*=1000 to the network of four nodes displayed in figure 2. The resulting point estimate 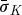 of the ranks for this network assigns ranks 1, 2, 3 and 4 to nodes *A*, *B*, *C* and *D*, respectively. However, owing to the sparsity of information about conflict outcomes in this example, the uncertainty of the ranking estimates is very high. This uncertainty can be observed in figure 5, which provides for each node a bar plot indicating the frequency of assignments of that node among each of the four possible ranks. Indeed, it would be unwise to confidently assert the true rank of any node here.

As a further illustration, we create an artificial dataset consisting of 20 subjects which are ranked linearly, with each subject assigned a true dominance potential greater than 0.5 over all lower-ranking subjects, which increases as the difference in ranks increases. We randomly select pairs to experience conflicts, and determine the outcome of each conflict stochastically based on the true dominance potential. Each subject experiences between 17 and 24 conflicts, so that sufficient data should be available to estimate well both the true ranking and the true dominance potentials using the Beta random field. Given that there are many cases in our generated data in which a lower-ranking subject won the conflict (31 out of 200), we expect some ranking error. The point estimate for the ranking coordinates after executing steps S1 and S2, with *K*=1000 and *R*=3, is

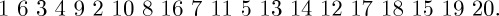
The ranks of nine subjects are estimated perfectly, while all but two are incorrect by no more than four positions. Hence, the overall ranking error is quite low. Looking instead at the estimates of the pairwise dominance potentials, we count the frequency with which the true dominance potential falls within the corresponding 90 per cent empirical CI, and find success in 95 per cent of the cases. On average, the point estimate of the dominance potential based on the mean field differs from the true dominance potential by 0.144. Hence, we have strong grounds for confidence in our ability to accurately predict the outcome of a new pairwise conflict based on past observations.

## 5. Analysis for real datasets

### (a) Ranking of college football teams

To illustrate our method, we analyse the games played among the 120 NCAA Division I-A college football teams in the United States on a week-by-week basis throughout the 15 weeks of the 2010 regular season. We base our indirect dominance odds calculations on dominance paths of orders one, two and three, since the network diameter is 4 while the average distance between nodes is 2.43 and the median distance is 3. Since the network is very sparse, we do not make the effort to ensure that all dominance paths are non-overlapping. As we discuss in the electronic supplementary material, this should only make the estimates more conservative, if it impacts them at all. For each week of the season, we construct a competition matrix based on all games played between Division I-A teams up through the previous week. In this matrix, *c*_*ij*_ denotes the number of games in which Team *i* has beaten Team *j* up to the current week of the season. We then generate *K*=100 realizations of the Beta random field, and for each realization we compute the direct and indirect dominance odds, form the enhanced dominance probability matrix, and obtain an optimal ranking order for the 120 teams, following the procedures described above. We also obtain the corresponding mean field and its optimal ranking order.

In table 1, we list the top 21 teams at the close of the 2010 regular season based on the computed optimal ranking order of the mean field. We also include the remaining four teams which received an official rank from the Bowl Championship Series (BCS), and provide the rank assigned by the BCS and the regular season record for games against other Division I-A teams. We observe considerable disagreement between some of our rank assignments and both the BCS rank assignments and the average computer ranks. For example, we give South Carolina a rank of 7 and Alabama a rank of 8, while their BCS ranks are 20 and 16, respectively. Meanwhile, although Florida State, Hawaii, West Virginia and Central Florida were among the top 25 BCS ranks, we place them far down the list. This disagreement demonstrates the importance of including indirect dominance odds via dominance paths when evaluating the relative strengths of teams.

**Table 1.** Top 21 teams for the 2010 regular season based on the Beta random field, plus the remaining four teams among the BCS top 25, with the regular season record for Division I-A games and the rank assigned by the BCS.

rank	team	record	BCS rank
1	Auburn	12–0	1
2	Oregon	11–0	2
3	TCU	11–0	3
4	Stanford	10–1	4
5	Arkansas	9–2	8
6	LSU	9–2	11
7	South Carolina	8–4	20
8	Alabama	8–3	16
9	Michigan State	10–1	9
10	Texas A&M	8–3	17
11	Oklahoma	11–2	7
12	Wisconsin	10–1	5
13	Ohio State	11–1	6
14	Nebraska	9–3	18
15	Oklahoma State	10–2	14
16	Utah	10–2	19
17	Mississippi State	7–4	21
18	Missouri	9–2	12
19	Nevada	11–1	15
20	Boise State	11–1	10
21	Virginia Tech	11–1	13
29	Florida State	8–4	23
44	Hawaii	9–3	24
45	West Virginia	8–3	22
57	Central Florida	9–3	25

During the first five weeks most components of 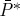 equal 0.5, since few games have been played, so that numerous mean-field rankings produce a minimum cost of zero. Hence, there is considerable uncertainty in the rankings. As the season progresses, the optimal value of the cost function when the rows and columns of the mean field 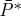 are permuted according to the computed optimal ranking increases steadily, reaching 22.89 by Week 10 and 30.34 by the end of the regular season. When the teams are arranged in an alphabetical order, which is the order prior to optimization, the computed cost of the corresponding mean field is 1267.29. Hence simulated annealing produces a dramatic reduction in the cost by finding an optimal ranking order. It should be noted that many permutations of the top ranks will produce the same minimal cost, since any pair of undefeated teams will always have a dominance probability of 0.5 in the mean field.

**Figure 6.**
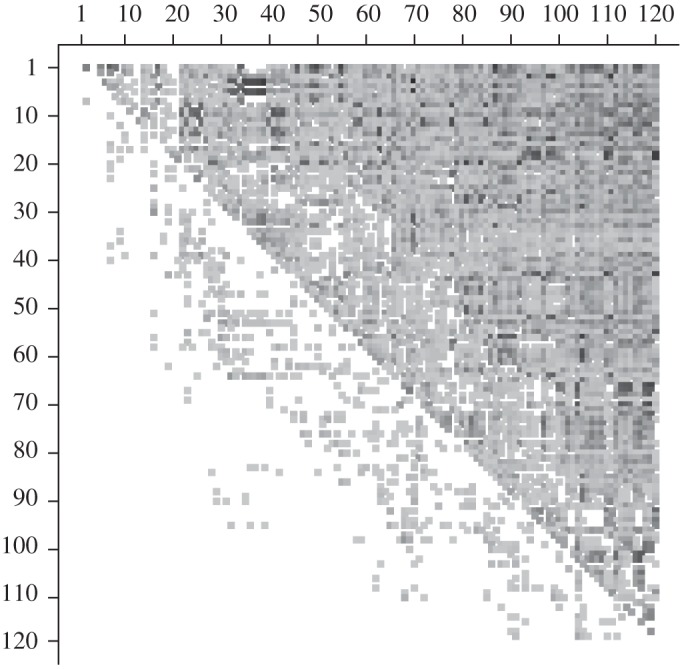
Optimized mean field. Graphical representation of the mean field 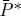 with its rows and columns in the computed optimal ranking order. Grey squares indicate cells containing values exceeding 0.5, with the darkness of a square signifying the magnitude corresponding value.

Figure 6 is a graphical representation of the mean field 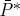 after its rows and columns are arranged in the optimal ranking order. Cell (*i*,*j*) is coded with a grey square if 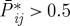, and is left blank otherwise. The darkness of the grey square represents the value of 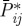, so that values close to 1 are dark grey and values close to 0.5 are light grey. Each grey square in the lower triangle indicates a lower-ranked team that dominates a higher-ranked team, while blank squares in the upper triangle signify both the counterparts to the grey squares in the lower triangle and cases in which neither team dominates the other. We observe that the majority of the grey squares in the lower triangle are light grey, and that they are concentrated near the diagonal. This concurs with our goal of finding a ranking order that minimizes the cumulative error. As discussed above, there are other ranking orders that also achieve this minimum, reflecting the true uncertainty in the rankings.

We may also use the components of the mean field computed for each week to help predict the outcomes of future games between teams. That is, we forecast that Team *i* will beat Team *j* if 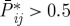. As the season progresses and more data become available, our accuracy should improve. To test this claim, we predict the outcomes of games for weeks 7–15 of the regular season based on the values in the matrix 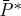 at game time, and then check our success rate. Prior to week seven most of these values are 0.5, so that no prediction is possible for the majority of games. Note that the only game in the 15th week is the Army–Navy game. In table 2, we display the number of successful predictions out of the total number of games for which a prediction is possible, with the corresponding success rate, for each week. The rate generally improves later in the season, with a 79 per cent success rate in week 14. Of course, it can be argued that the outcomes of most of these games were not surprising to college football fans, and that the ones for which we were unable to make a prediction are the ones for which an accurate prediction would be most impressive. Neverthless, the reasonably high success rates are confirmation of the usefulness of our approach.

**Table 2.** Weekly success rates for predicting game outcomes.

	successes	total	rate
week 7	12	22	0.545
week 8	20	30	0.667
week 9	28	41	0.683
week 10	28	43	0.651
week 11	34	54	0.630
week 12	39	53	0.736
week 13	32	50	0.640
week 14	15	19	0.789
week 15	1	1	1.000

### (b) Ranking of rhesus macaques

**Figure 7.**
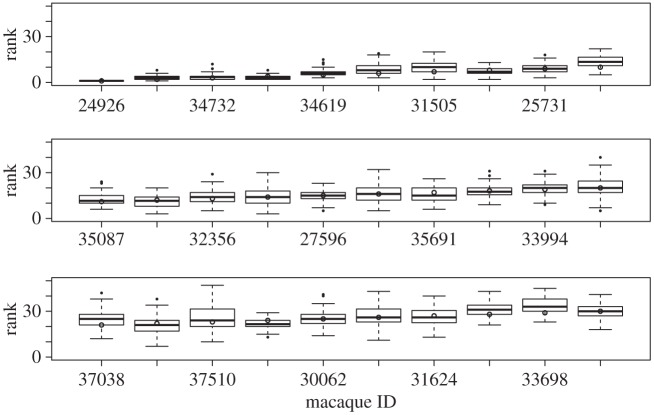
Cage 5 rankings, with uncertainty. Top 30 macaques in Cage 5, according to their estimated rank coordinates (signified by the small circles), with boxplots indicating the uncertainty of the individual coordinate estimates.

We apply our computing algorithm to conflict outcome data collected on a captive society of rhesus macaques housed at the CNPRC. These behavioural data were collected between June 2008 and April 2009, and include all observed aggressive interactions that had a decisive outcome (for detailed methods, see Beisner *et al.* 2010). For example, an interaction where an initiator threatens a recipient and the recipient runs away is counted as a win for the initiator and a loss for the recipient. Other aggressive behaviours include lunging, chasing and biting. The group in Cage 5, which was analysed in Fushing *et al.* (2011), using an earlier version of the current approach, includes 94 adult rhesus macaques. We obtain an estimated ranking coordinate for each macaque, along with a distribution of rank estimates, based on the outcomes of each of *K*=100 iterations of our algorithm. In figure 7, the top 30 macaques are listed according to their estimated rank coordinates, based on their ID numbers, along with a boxplot that indicates the uncertainty of that rank estimate. The top-ranking macaque is the alpha male (ID 24926), whose position in the hierarchy is virtually certain. The Beta male (ID 22898) occupies the second position with very little uncertainty. The rank coordinates for the next 28 macaques (mostly females) are less certain, based on the application of our method to the available data. The simulated annealing algorithm arrives at coordinate assignments that result in a reduction in the cost from initial values in the range 333–505 to optimal values in the range 209–359, with an average reduction of 138. However, when the mean field is formed, simulated annealing reduces the cost from an initial value of only 22.44 to an optimal value of 11.98. Given the uncertainty in the data, this low value provides strong evidence of the general accuracy of the estimated ranking of the 94 subjects.

Macaque societies are organized according to matrilines. Based upon the optimal ranking network, we list in table 3 the estimated rank coordinates for the adult members of each matriline in the Cage 5 enclosure. Males are distinguished by bold italics. The most significant finding from this analysis is that the ranking network for this macaque society seems to have a ‘governing’ group consisting of a dominant matriline (C7) subordinate only to the alpha non-related male (NRM) and Beta (M10) males. This dominant matriline is at the top of a hierarchy of matrilines, some of which appear to be on the same tier (e.g. matrilines X1 and S16). There are also several elite individuals of either gender from several lower-ranking matrilines, such as the high-ranking male from matriline Z2. Our results agree well with the expectations of the primatologists at the CNPRC. This sophisticated structure may be one of the key factors underlying the group's stability.

**Table 3.** Summary of ranking coordinates for rhesus macaques in Cage 5 grouped according to matrilines.

matriline	ranking coordinates
NRM	***1***
C7	3 4 5 6 7 10 11 ***14*** 17 ***21*** 23
X1	9 13 18 19 26 ***87 91*** 92 ***94***
S16	15 24 25 ***30 36*** 65 ***67***
J6	28 31 32 44
L4	16 29 ***40***
D10	12 ***42***
F10	20 22 27 33 34 35 38 43 45 ***48 50*** 51 ***55*** 56 ***59 69*** 73
N4	39 41 46 53 54 57 58 ***60*** 61 66 70 ***74 76***
M10	***2*** 37 47 ***62 78 81***
Z2	***8*** 49 52 63 68 ***72*** 75 77 79 80 ***82*** 83 84 85 86
G8	***64*** 71 88 89 90 93

## 6. Discussion

We show the impropriety and counter-intuitivity of MLE in the task of constructing a nonlinear ranking network. In contrast, we present an algorithmic network ranking approach that incorporates both direct and indirect sources of local information in the data, and provide a basis for conditional statistical inference that addresses the inherent uncertainty in computing dominance potential. From both aspects, our approach is rather unconventional, but very effective. The idea of a Beta random field on a graph is proper and essentially informative. It is also new. The conditional statistical inference is a brand-new way to analyse data represented by a graph or network. The computational load is very economical for even a very large number of nodes.

The perspective of incorporating indirect information extracted from the empirically observed dominance paths is important in our development. The induced transitive dominance odds coupled with the direct dominance odds make our analysis informative and effective. In contrast, MLE and Bayesian analysis suffer from severe computational complexity and become very unreliable even for a network having as few as 20 nodes (Adams 2005). As a consequence, the compromising nature of the likelihood approach can further cause severe bias in MLE-based ranking, whereas this approach is essentially bias-free.

Our analysis of real datasets from the NCAA and the CNPRC brings out remarkable results that illustrate the range of usefulness for this approach, from improving the legitimacy of the rankings for a variety of sports teams to enhancing biologists' understanding of the structural network in societies of primates and other social species. We anticipate that this ranking approach will provide a powerful scientific tool for computing more reliable ranking networks and resolve many potential disputes in diverse scientific fields and real-world applications.
